# The LRRC8A Mediated “Swell Activated” Chloride Conductance Is Dispensable for Vacuolar Homeostasis in Neutrophils

**DOI:** 10.3389/fphar.2017.00262

**Published:** 2017-05-12

**Authors:** Philippe Behe, Juliet R. Foote, Adam P. Levine, Craig D. Platt, Janet Chou, Fernando Benavides, Raif S. Geha, Anthony W. Segal

**Affiliations:** ^1^Division of Medicine, University College LondonLondon, UK; ^2^Division of Immunology, Boston Children’s Hospital, Harvard Medical School, BostonMA, USA; ^3^Department of Epigenetics and Molecular Carcinogenesis, The University of Texas MD Anderson Cancer Center UTHealth Graduate School of Biomedical Sciences, HoustonTX, USA

**Keywords:** neutrophil, vacuolar homeostasis, NADPH oxidase, ion channel, chloride, VSOR Cl^-^ current, LRRC8A, Tamoxifen

## Abstract

The dialysis of human and mouse neutrophils in patch clamp experiments in the conventional whole-cell mode induces the emergence of a chloride (Cl^-^) current that appeared to be primarily regulated by cytoplasmic ionic strength. The characteristics of this current resembled that of the classical, and ubiquitous volume-sensitive outwardly rectifying Cl^-^ current: strong outward rectification, selectivity sequence of the Eisenman1 type, insensitivity to external pH and strong inhibition by tamoxifen, DCPIB and WW781. We show that this current is essentially supported by the leucine rich repeat containing 8 A (LRRC8A); the naturally occurring LRRC8A truncation mutant in *ebo/ebo* mice drastically reduced Cl^-^ conductance in neutrophils. Remarkably, the residual component presents a distinct pharmacology, but appears equally potentiated by reduced ionic strength. We have investigated the role of the LRRC8A-supported current in the ionic homeostasis of the phagosomal compartment. The vacuolar pH, measured using SNARF-1 labeled *Candida albicans*, normally rises because of NADPH oxidase activity, and this elevation is blocked by certain Cl^-^ channel inhibitors. However, the pH rise remains intact in neutrophils from the *ebo/ebo* mice which also demonstrate preserved phagocytic and respiratory burst capacities and normal-sized vacuoles. Thus, the LRRC8A-dependent conductance of neutrophils largely accounts for their “swell activated” Cl^-^ current, but is not required for homeostasis of the phagosomal killing compartment.

## Introduction

Polymorphonuclear granulocytes, or neutrophils, are the most common of the white blood cells and are primarily responsible for the killing and digestion of invading bacteria and fungi which they engulf into an invagination of the plasma membrane known as the phagocytic vacuole. The NADPH oxidase passes electrons from NADPH across this membrane onto oxygen to generate superoxide and hydrogen peroxide in the vacuole. This process is electrogenic and the charge generated across the wall of the vacuole must be compensated, by the passage of cations into, and/or of anions out of, the vacuole for electron transport to continue. The movement of protons through the proton channel HVCN1 into the vacuole accounts for the bulk of this charge compensation. However, ions other than protons must be involved because there is a net consumption of protons in the vacuole, as evidenced by an elevation of pH in this compartment to about 9.0 (Segal et al., 1981; Levine et al., 2015).

Cl^-^ appears to play an important role in this process because certain Cl^-^ channel blockers inhibit this elevation in pH (Foote et al., 2017). There are two competing theories as to the direction of the movement of Cl^-^. On the one hand, it is believed that the efflux of Cl^-^ through the proton antiporter CLCN3 plays a role in compensating the oxidase-induced depolarization of the vacuolar membrane (Moreland et al., 2006). The alternative theory is that Cl^-^ has to enter the vacuole to be oxidized to microbicidal hypochloric acid (HOCl) by myeloperoxidase, and both the CFTR channel (Painter et al., 2006; Aiken et al., 2012) and KCC3, a K^+^-Cl^-^ co-transporter (Sun et al., 2012), have been implicated in this process (Klebanoff et al., 2013; Green et al., 2014).

The neutrophil is richly endowed with Cl^-^ transporters, exchangers, antiporters and channels that orchestrate active transport, exchange or electro-diffusion. These include anion exchangers (Simchowitz and De Weer, 1986), Na^+^/K^+^/2Cl^-^ co-transporters (Bialasiewicz et al., 2004), and calcium- and swell-activated channels (Krause and Welsh, 1990; Schumann et al., 1993; Stoddard et al., 1993). Amongst the diffusive pathways available to Cl^-^ in neutrophils; the evidence of a large Cl^-^ efflux (Shimizu et al., 1993; Busetto et al., 2007), the swelling of the phagocytic vacuole (Reeves et al., 2002; Levine et al., 2015), and the recent molecular identification of LRRC8A as an indispensable component of the volume-regulated anion channel (VRAC) or volume-sensitive outwardly rectifying (VSOR) current (Qiu et al., 2014; Voss et al., 2014) prompted us to consider this channel as a possible participant in compensation of the current generated by the oxidase.

In whole cell patch clamp experiments on neutrophils, we observed a Cl^-^ current that was activated upon dialysis of the cell that follows breakthrough. Its properties included a primary sensitivity to ionic strength and a strong inhibition by tamoxifen, typical of the VRAC currents (Valverde et al., 1993; Voets et al., 1999). To identify this current we studied *ebo/ebo* mice which have a frameshift mutation in exon 3 of *LRRC8A*, resulting in dramatically reduced VRAC activity (Platt et al., 2017). We found this large Cl^-^ current to be missing from *ebo/ebo* mouse neutrophils. A small residual Cl^-^ current was detectable that was potentiated by low ionic strength but, unlike the VRAC, it was insensitive to tamoxifen.

Having identified LRRC8A as a component of the channel producing the predominant Cl^-^ current we could identify in neutrophils, we examined the effect of its loss in the *ebo/ebo* mouse on NADPH oxidase activity and on vacuolar pH and swelling; parameters that are dependent upon the flux of charge compensating ions. We found all three parameters to be unaffected in *ebo/ebo* mice.

## Materials and Methods

### Ethical Statement

All animal work was conducted with the license and approval of the United Kingdom Home Office (Project license 70/8452). Human participation in this research was approved by the Joint UCL/UCLH Committees on the Ethics of Human Research (Project number 10/H0806/115). All participants provided informed consent in accordance with the Declaration of Helsinki.

### Cell Isolation

Human peripheral blood neutrophils were purified by dextran sedimentation, centrifugation through Lymphoprep and hypotonic lysis. Isolated neutrophils were kept on ice in a Ca^2+^-free extracellular buffer or phosphate buffered saline and used over the following 6 h.

Mouse neutrophils were obtained from femoral bone marrow or circulating blood (by cardiac puncture) after sacrifice of the animal by asphyxia and cervical dislocation. Neutrophils were purified by negative selection, using a column-free magnetic separation protocol (Mouse neutrophils enrichment kit 19762, Stem Cell technologies, Cambridge, UK). Negatively selected mouse bone marrow cells were found to be easier to patch and remained usable for much longer than human cells isolated as described above. Mouse neutrophils were kept on ice in a Ca^2+^-free extracellular buffer, and used for up to 30 h after purification.

### Electrophysiological Recordings and Solutions

All chemicals were obtained from Sigma or Tocris.

Recordings were obtained in the whole cell mode of the patch-clamp technique, using a Cairn Optopatch amplifier under control of John Dempster’s software WinWCP3.9.0 (University of Strathclyde). Under voltage clamp, command voltages consisted in either linear ramps depolarizing the membrane from -120 to +100 mV in 2.6 s or in series of depolarizing voltage steps, 500, 750, or 1000 ms long, from -120 to +100 mV in 20 mV increments and at 2 s intervals. Control and drug containing solutions were gravity-fed into a 100 μl chamber containing adherent neutrophils via an 8-valve dispenser (ALA-VM8, ALA Scientific Instruments, Farmingdale, NY, USA) at a rate of ∼0.5 mL/min.

To minimize variations in junction potential when the extracellular Cl^-^ concentration was varied, the ground electrode was a 3M KCl agar bridge. To prevent alteration in the bath potassium concentration, the bridge and the outflow line were both set in a separate chamber away from the main pool. The potentials cited are corrected for junction potentials calculated with the application available in the Clampex software (Axon Instruments, Molecular Devices, Sunnyvale, CA, USA). The pipettes were about 2 to 3 MegaOhm when filled with a conventional internal solution (containing 140mM KCl).

The osmolarities of the solutions were checked with a Löser freezing point osmometer (Camlab House, Cambridge, UK). Except where specified, internal and external solutions were, respectively, set in the 280–290 mOsm and 300–310 mOsm ranges.

Ionic strength was calculated as half the weighted sum of all individual ion concentrations (*c*_i_), where the weight for each species is the square of its valence (*z*_i_):

$$\text{Is}=0.5\;\left(\Sigma c_{i}z_{i}^{2}\right)$$

Initial observations were made with unselective internal and external solutions, the former being of standard or increased osmolarities. To isolate Cl^-^ currents, internal and external solutions were subsequently either Cesium- or NMDG-based, the latter excluding the participation of unselective cation conductances known to be expressed by human and murine neutrophils (Heiner et al., 2003). These solutions were of standard osmolarity (Int 280mOsm/ext 300mOsm) but the internals of variable ionic strengths. **Table 1** gives the composition, osmolarities and ionic strength of sets of paired intracellular/extracellular solutions, as used. To compare the Cl^-^ conductance when using sets of internal/external solutions that defined different reversal potentials (as in **Figure 4**), the currents were normalized to the corresponding junction potential corrected Cl^-^ driving forces.

**Table 1:** Intracellular and extracellular solutions for patch-clamp recordings

SetA: Unselective solutions, hyperosmotic internal **Figure 1**) *E*_Cl_: -11 mv, ΔOsm: 50 mOsm .		Set B: Unselective solutions, standard internal (**Figures 2**, 3) *E*_Cl_: -84 mv, ΔOsm: -10 mOsm .	
INTERNAL	EXTERNAL	INTERNAL	EXTERNAL
90 mM KCl	135 mM NaCl	27 mM K Aspartate	140 mM NaCl
36 mM K Gluconate	4.7 mM NaOH	100 mM K Gluconate	5 mM KCl
13 mM KOH	5 mM KCl	13 mM KOH	1 mM MgCl_ 2_
5 mM NaCl	0.5 mM MgCl_2_	5 mM NaCl	1.5 mM MgCl_2_
0.9 mM CaCl_2_	1.8 mM CaCl_2_	0.2 mM CaCl_2_	10 mM Hepes
2 mM EGTA	10 mM Hepes	1 mM EGTA	5 mM Glucose
10 mM Hepes	5 mM Glucose	20 mM Hepes	
3 mM Mg ATP		2 mM Mg ATP	
5 mM NADPH			
pH 7.35	pH 7.35	pH 7.35	pH 7.35
Osm 350 mOsm	Osm 300 mOsm	Osm 290 mOsm	Osm 300 mOsm
Ionic strength 144 mM	Ionic strength 149 mM	Ionic strength 144 mM	Ionic strength 155 mM

**Set C: Cesium based solutions *E*_Cl_: -26 mv, ΔOsm: -20 mOsm** .		**Set D: NMDG based solutions (**Figure 4**) *E*_Cl_: -32 mv, ΔOsm: -10 mOsm** .	
**INTERNAL**	**EXTERNAL**	**INTERNAL**	**EXTERNAL**

Low strength		Standard strength	
44 mM CsCl	2 mM NaCl	155 mM NMDG	140 mM NMDG
30 mM CsOH	132 mM CsCl	32 mM HCl	133 mM HCl
2 mM NaATP	6 mM CsOH	105 mM Gluconic acid	2 mM CaCl_2_
2 mM MgCl_2_	1 mM MgCl_2_	4 mM EGTA	1 mM MgCl_2_
1 mM CaCl_2_	2 mM CaCl_2_	1 mM CaCl_2_	10 mM Hepes
10 mM Hepes	10 mM Hepes	10 mM Hepes	5 mM Glucose
4 mM EGTA	5 mM Glucose	2 mM MgCl_2_	
157 mM Sucrose		2 mM MgATP	
pH 7.35	pH 7.35	pH 7.35	pH 7.35
Osm 280 mOsm	Osm 300 mOsm	Osm 290 mOsm	Osm 300 mOsm
Ionic strength 84 mM	Ionic strength 143 mM	Ionic strength 154 mM	Ionic strength 148 mM

		**Set E: NMDG based solutions (**Figures 4**, 5) *E*_Cl_: -32 mv, ΔOsm: -20 mOsm**.
		
		**INTERNAL EXTERNAL**	

		Low strength	
		50 mM NMDG	140 mM NMDG
		32 mM HCl	133 mM HCl
		4 mM EGTA	2 mM CaCl_2_
		1 mM CaCl_2_	1 mM MgCl_2_
		2 mM MgCl_2_	10 mM Hepes
		10 mM Hepes	5 mM Glucose
		2 mM MgATP	
		180 mM Sucrose	
		pH 7.35	pH 7.35
		Osm 280 mOsm	Osm 300 mOsm
		Ionic strength 53 mM	Ionic strength 148 mM

Some previous reports concerning outward Cl^-^ currents in neutrophils could be due to contamination by the large proton current that these cells demonstrate (Schumann and Raffin, 1994). We took advantage of the availability of HVCN1^-/-^ mice to exclude such uncertainty. When working on other knock-out (KO) mice, or on human cells, protons current were repressed with 100 μM zinc (Zn^2+^), a dose that is not expected to substantially affect phagocytosis (Yatsuyanagi et al., 1987; Morgan et al., 2009). When Zn^2+^ was added to gluconate containing solutions, its concentration was increased to preserve that of unbound Zn^2+^. The concentrations of free divalent ions were estimated using the MaxChelator program written by Chris Patton.^1^

### Imaging

To measure the variation in cell volume that accompanied the onset of whole cell recording, images were acquired at intervals using a Pixel Fly camera^2^ and the Camware software driven by winWCP. They were analyzed in 2 dimensions using Image J.^3^

Confocal imaging: The pH of individual vacuoles formed by phagocytosis of opsonised SNARF1-labeled *Candida albicans* or *Candida* was measured by confocal ratiometric fluorescence microscopy as described (Levine et al., 2015). Where antagonists of Cl^-^ transport pathways were used, cells were preincubated for 5 min in medium containing the inhibitors before being exposed to *Candida*, and antagonists were present in the medium for all of the recorded experiment. Consequently, both the vacuolar and extracellular media contained the drug under investigation. Images were acquired at 25 to 35 min after addition of *Candida*. Our vacuolar pH measurements were end-point determinations and were not synchronized to particle uptake as previously described. The SNARF fluorescence ratios were calibrated to pH values using standard curves derived from separate calibration experiments as described by Levine et al., 2015.

### Phagocytosis

Phagocytosis was evaluated on the confocal images as described (Levine et al., 2015). The proportion of phagocytosing cells was evaluated over a randomly selected area comprising 100 cells and this fraction normalized to the ratio of *Candida* to cells. A phagocytic index was defined as the product of the normalized proportion of phagocytosing cells by the mean number of *Candida* ingested by each of them.

### Respiratory Burst

The respiratory burst of Wild-type or *ebo/ebo* bone marrow neutrophils was measured using the Amplex Ultra assay (Life technologies) which monitors the extracellular production of H_2_O_2_ after stimulation of the cells with PMA (3 μM).

### Statistical Analysis

Paired *t*-tests were used to assess the significance of the effects of inhibitors on oxygen consumption levels. Differences in vacuolar pH (fluorescence ratios) were validated in individual experiments by Wilcoxon rank sum test, difference between means of replicates checked with paired *t*-tests. Sample sizes are given in the figure legends to figures.

## Results

### A Cl^-^ Current Run Up upon Dialysis of Human Neutrophils

To investigate the capacity of swell activated currents to control the membrane potential of activated neutrophils, we supplied human neutrophils with a hyperosmotic (350 mOsm), NADPH-containing and physiological internal solution in the conventional whole cell mode. On breakthrough, the majority of cells (5/7) appeared variously depolarised (mean +30 mV, range +10/+63 mV) by a largely linear current which we attribute to the activity of the NADPH oxidase. With a delay, a rectifying outward current developed on this background which soon efficiently counteracted the depolarising oxidase activity.

**FIGURE 1:**
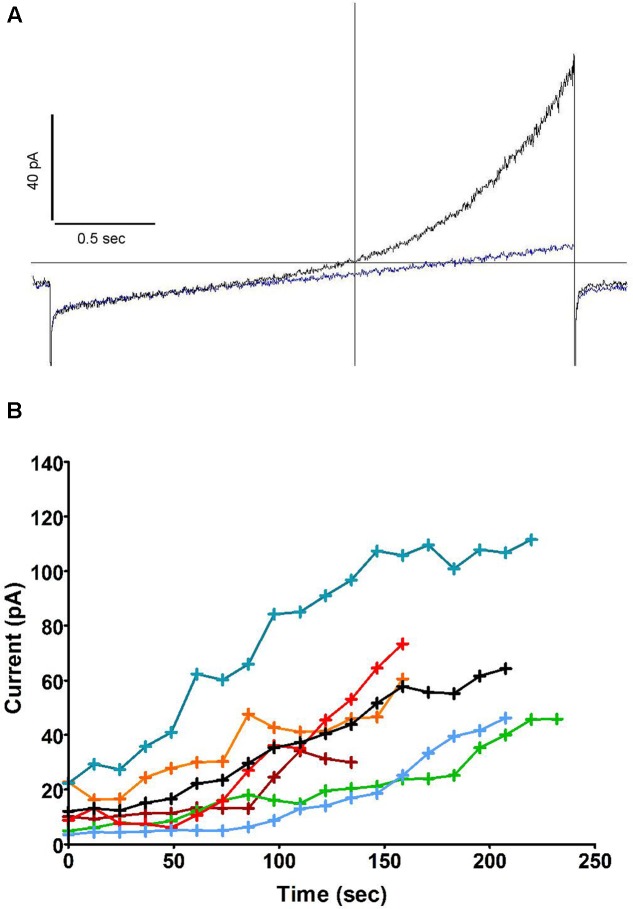
**An outward current develops after breakthrough and counteracts the oxidase induced depolarisation**. **(A)** Whole cell current in human neutrophils in response to depolarising 2.6 s long voltage ramps from –127 to +93 mV at 48 s (blue trace) and 160 s (black trace) after breakthrough (holding potential: –87 mV). **(B)** Time course of the outward current increase measured at +93 mV (i.e., at the top of voltage ramps such as shown in **A**) in 6 cells using solutions from set A (**Table 1**). The average trace is in black.

**Figure 1A** is a typical example of these early observations. Note that the emergence of this outward current is not associated with slow tail currents on return to negative potential (-30 mV), as one would expect from proton currents (*E*_H_^+^ = 0 mV). The time course of the outward current increase in six cells patched in the same conditions is shown in **Figure 1B**, the black trace represents the average change in current of the six cells.

**FIGURE 2:**
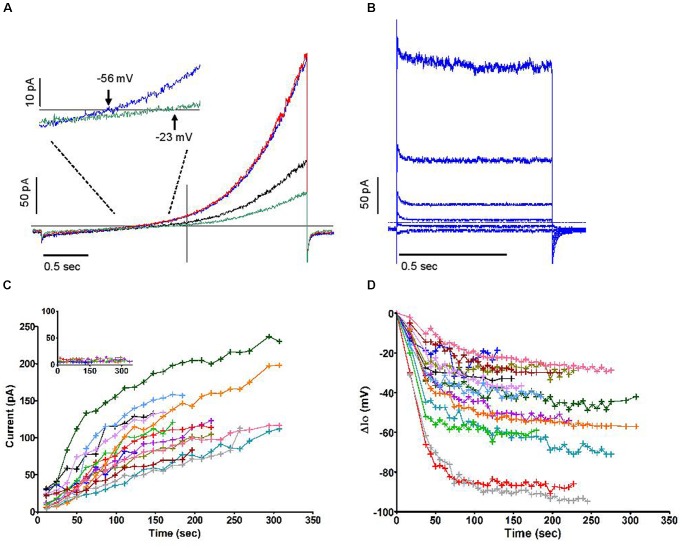
**The outward current is carried by Cl^–^ and is insensitive to Zn^2+^**. **(A)** Superimposed human neutrophil whole-cell currents in response to depolarising voltage ramps (from –136 to +84 mV) at 36 s (black trace) and 270 s (blue trace) after breakthrough. A steady state level of outward current was then attained which is not affected by Zn^2+^ 100 μM (red trace) but depends on external Cl^–^ (green trace). The inset focuses on the change of reversal that accompanies the substitution of external Cl^–^ for gluconate. **(B)** Superimposed responses to stepped voltage pulses (from –96 mV to –136, –76, –36, +4, +44 and +84 mV, respectively, from bottom to top traces) in control conditions after full development of the emerging outward current. **(C)** Time course of outwardly rectifying Cl^–^ current increase in 14 human neutrophils at +84 mV under iso-osmotic conditions. Inset: very minimal current was recorded in perforated whole cell conditions. **(D)** Time course of plasma membrane hyperpolarizations (ΔIo) in 14 human neutrophils (the same cells as **C**) after the development of the outwardly rectifying Cl^–^ current under iso-osmotic conditions. All experiments were carried out using solution set B (**Table 1**).

Further experiments (**Figure 2A**) showed that this outward current also emerged when recording from cells dialysed with a pipette solution of standard osmolarity (300 mOsm) and did not depend on oxidase activity, as the same experiment repeated in solutions lacking NADPH did not prevent its development (the same current was observed in neutrophils prepared from gp91phox^-/-^ (CGD) mice, not shown). The current was insensitive to Zn^2+^ but drastically reduced by the substitution of Cl^-^ for external gluconate. Interlaced with ramps, stepped voltage commands revealed its fast activation and deactivation kinetics with no appreciable tails, yielding quasi-steady current along 0.5 s pulses (**Figure 2B**). The top current, however, at +84 mV, was not quite sustained: it slowly decreased during the pulse. **Figure 2B** illustrates a marginal case of such deactivation or “sag.” The amplitudes of that “sag,” measured as the loss of current between start and end of the depolarizing pulse, and the threshold of its occurrence, were observed to vary substantially from cell to cell: it is likely that parameters other than the channel itself are contributing to that feature. We have noticed its amplification in a number of conditions discussed elsewhere in this report. **Figure 2C** represents the time course of outward current increase at +84 mV in that cell and in others under the same conditions. Average run up time constant was 92 s toward an extrapolated value of 124 pA on average. In the absence of NADPH, the developing current will draw the cell potential in the negative range, well below the equilibrium potential of protons. The range of these hyperpolarizations is shown in **Figure 2D**. Their magnitude (mean amplitude -46.3 mV), in conditions that do not restrict the membrane conductance, emphasizes the potential significance of this current in the control of the neutrophil membrane potential.

**FIGURE 3:**
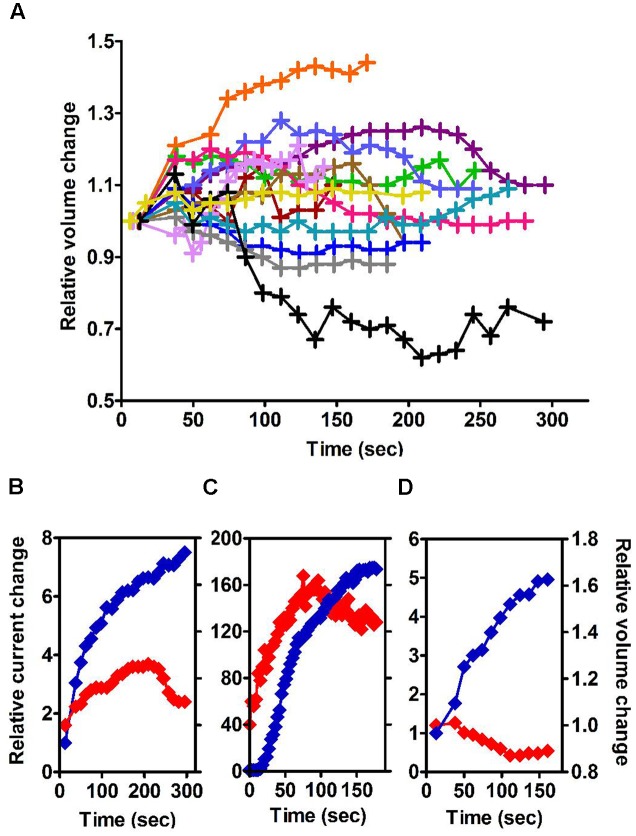
**(A)** Time courses of change in cell volume following the establishment of whole-cell recording conditions under iso-osmotic conditions in 13 human neutrophils carried out in solution set B (**Table 1**). **(B–D)** Three examples of clear divergence between relative changes in volume and current amplitudes. All measures are normalized to their initial values. Blue traces represent the relative current change (left *y* axis). Red traces represent the relative volume change (right *y* axis). Experiments for **(B,D)** used human neutrophils and were carried out in solutions set B, while solution set C was used for **(C)** and was recorded in a mouse neutrophil (**Table 1**).

Similar current profiles, with or without a “sag,” have been observed in various cell types for currents deemed to be “swell activated” (Nilius et al., 1997). Although the majority of cells had a tendency to swell moderately after being dialysed with an iso-osmotic internal solution (**Figure 3A**, pooled time course of cell areas), in which case the swelling accompanied the rise in outward current, our data do not support a causal relationship between these phenomena. A number of observations contribute to disconnect them functionally, some of which are illustrated in **Figure 3**: swelling phases were often reversed with no loss of the current that had emerged (**Figure 3B**); cells that swell did so immediately after breakthrough while a delay can be resolved in the current increase (**Figure 3C**, obtained from a murine cell); the current was seen to rise in cells that shrunk (**Figure 3D**). Overall the correlation coefficient between the time course of cell volume (inferred from measurements of the cell’s perimeter) and that of peak current was 0.08. This suggests that membrane stretch, as is assumed to occur during swelling, is not the primary cause of the increase in outward current. Accordingly, imposing a direct mechanical stretch to the membrane using the patch pipette did not trigger a current similar to the one described here. We also noted that when subject to positive or negative pressure in the “on cell” mode, the membrane of neutrophils did not yield volleys of stretch-sensitive channel activity as can be seen in other cell types (Yang and Sachs, 1990). Independently of membrane stretch or curvature, a current that accompanies swelling may be expected to respond to the dilution of the intracellular media. Later in this report, we document an aspect of this, i.e., the influence of reduced ionic strength. More specifically, the dilution of an internal repressor of the channel remains a plausible mode of activation upon breakthrough. The slow reversion of current amplitude often observed after its peak would furthermore imply that multiple factors are governing it.

### Perforated Whole Cell Recordings

The Cl^-^ conductance of resting neutrophils is known to be very low (Simchowitz and De Weer, 1986). Consistent with its negligible amplitude measured immediately after breakthrough, and suggesting that the channel may indeed be under the control of a diffusible repressor of large molecular weight (>300), we recorded no current in the perforated mode (see inset in **Figure 2C**). As we verified optically by the reduction of nitroblue tetrazolium (Schrenzel et al., 1998) and monitored in current clamp while perforation by amphotericin proceeded, neutrophils patched in that mode often activated spontaneously. Each of the six cells whose currents are shown in **Figure 2C** (inset) had in fact demonstrated transient oxidase-driven depolarisations averaging +61 mV (in the presence of 100 μM Zn^2+^). Yet subsequent voltage clamp recording revealed no substantial outward current. Their oxidase-driven depolarisation was amplified by Zn^2+^, but insensitive to the substitution of external Cl^-^ with gluconate (with free Zn^2+^ concentration preserved). It appears that plasma membrane-based oxidase activity is not dependent on Cl^-^ influx and does not, at least in the case of these spontaneous activations, induce a substantial Cl^-^ conductance. We have independently observed that millimolar concentrations of H_2_O_2_ in the external media were without effect (data not shown).

Having verified the potential significance and basic nature of this current, we have characterized it further using more selective solutions. As its molecular identification was foreseen to require the use of KO mice, these additional experiments were carried out using mouse neutrophils which were found to express a similar current. In association with selective solutions, HVCN1^-/-^ neutrophils allowed the observation of quasi-pure Cl^-^ currents without using Zn^2+^.

### Mouse Neutrophils Express Large Outwardly Rectifying Cl^-^ Currents in Whole Cell

Patching mouse neutrophils in the conventional whole cell conformation induced a dominating Cl^-^ conductance. Using selective (NMDG-based) external and internal solutions of physiological ionic strength, the outward current rose from barely 1 to 58.6 pA on average at +100 mV within about 5 min of breakthrough (time to 50%: 150 s).

**FIGURE 4:**
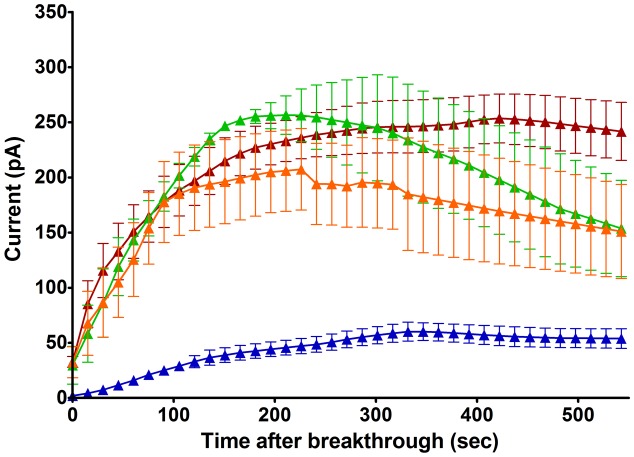
**Time course of emerging outward Cl^–^ currents at +100 mV in mouse neutrophils (HVCN1^–/–^ and WT cells treated with Zn^2+^ 100 μM), normalized to Cl^–^ driving force (**Table 1**), with selective internal solutions of standard or reduced ionic strength and selective external solutions**. Blue trace: Untreated cells, NMDG Cl^–^ based, standard ionic strength internal solutions [Solutions set D (**Table 1**), *n* = 4]. Brown trace: Untreated cells, NMDG Cl^–^ based, low ionic strength internal solutions [Solutions set E (**Table 1**), *n* = 5]. Green trace: cells treated with PMA (1 μM), NMDG Cl^–^ based, low ionic strength internal solutions (*n* = 3). Orange trace: cells treated with FMLP (1 μM) and Cytochalasin B (5 μg/ml), NMDG Cl^–^ based, low ionic strength internal solutions, (*n* = 8). The errors bars are standard error of the mean.

As it did not convincingly appear that a mechanical stimulus, including swell-induced membrane stretch, was the primary determinant of this current (see above), we tested the influence of ionic strength, another parameter affected by cell swelling, at constant osmolarity and Cl^-^ driving force. With NMDG based solutions of reduced strength, in which ions contributed to 36% of the osmolarity, the outward Cl^-^ current emerged faster and reached higher amplitudes (223 pA within 3.5 min, **Figure 4**). Negatively selected mouse neutrophils provide much longer recordings than conventionally prepared human cells: it became clearer that a slow and quasi-linear run down phase followed the fast initial run up of the current. After reaching its peak value, current was lost at a rate of about 2.6% per minute.

As is commonly the case for “swell activated” currents, and as we observed on human neutrophils (**Figure 2B**), there was no resolvable time dependence in the channel activation by depolarising voltage pulses. The current that developed upon dialysis of the cell interior activated and deactivated very quickly, providing no “tails” to determine the voltage dependence of its conductance. In conditions that isolate the current rigorously, an activation curve can, however, be proposed on the basis of current and driving force values. Recordings made on the HVCN1^-/-^ background with tight seals and selective (NMDG-based) solutions showed that in standard, as well as in low, ionic strength conditions the threshold of activation is around -50 mV, and there was no sign of saturation at +90 mV.

**FIGURE 5:**
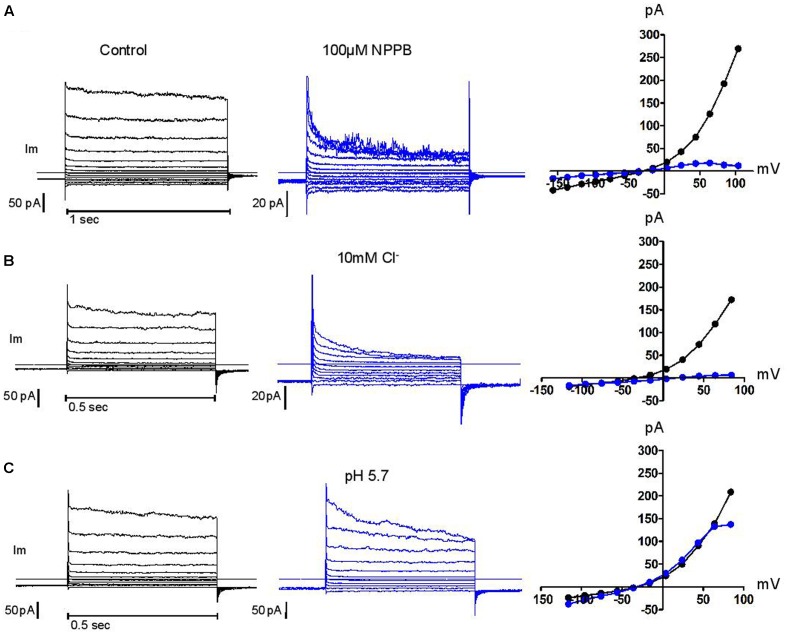
**Control of current inactivation in HVCN1^–/–^ neutrophils by NPPB 100 μM, a Cl^–^ channel inhibitor (A)**, reduced Cl^–^ concentration (10 mM, **B**) and low external pH (pH 5.7, **C**): each emphasizes the inactivation of the outwardly rectifying Cl^–^ current at high membrane potentials. Experiments were carried out in solution set E (**Table 1**).

Above +90 mV, the current was often seen to “sag” or inactivate within the duration of the depolarising pulse as was the case for human neutrophils. Failure to recover from that inactivation between successive voltage steps of increasing amplitudes will lead to overlapping current traces and results in the appearance of a region of negative conductance at the top end of current/voltage plots. In other cell types, external magnesium has been shown to be partly responsible for this inactivation, shared by many, but not all, Cl^-^ channels that are classified as swell-activated (Anderson et al., 1995; Braun and Schulman, 1996). While we found magnesium unable to induce or amplify this trait, we confirmed the capacity of acidic external pH, of blockers [5-nitro-1-(3-phenylpropylamino)benzoic acid, NPPB], tamoxifen, flufenamic acid (FFA), and of reduced external Cl^-^ concentrations, to do so and in a cumulative manner. Representative examples of these modulations are shown in **Figure 5**. The influence of these four parameters has been carefully documented (Voets et al., 1997) on BC3H1 myoblasts, together with their effect on recovery from inactivation. More recently, the molecular determinants of inactivation have been investigated on recombinant LRRC8 heteromers (Ullrich et al., 2016). While clearly involved, the heteromeric nature of the complex may not fully account for the variability of this phenomenon. We have observed inconsistency between neutrophils.

Even under physiological salt conditions, the dialysis-activated Cl^-^ current dominates other currents expressed by mouse neutrophils in conventional whole cell recordings: when using Cl^-^ free solutions to investigate cationic currents, NPPB was necessary to erase the residual highly sagging anionic current carried by the substitute gluconate (data not shown).

### Effect of pH

Considering the capacity of the NADPH oxidase to rapidly build large gradients of protons across the plasma or vacuolar membrane, pH regulation is a challenge for neutrophils which may respond by enabling pH dependent ion fluxes. Cl^-^ currents have been described that are subject to such regulation (Nobles et al., 2004; Lambert and Oberwinkler, 2005). In addition, a whole subfamily of Cl^-^ channels are Cl^-^/H^+^ antiporters (Stauber et al., 2012), and as such may directly participate in pH control.

**FIGURE 6:**
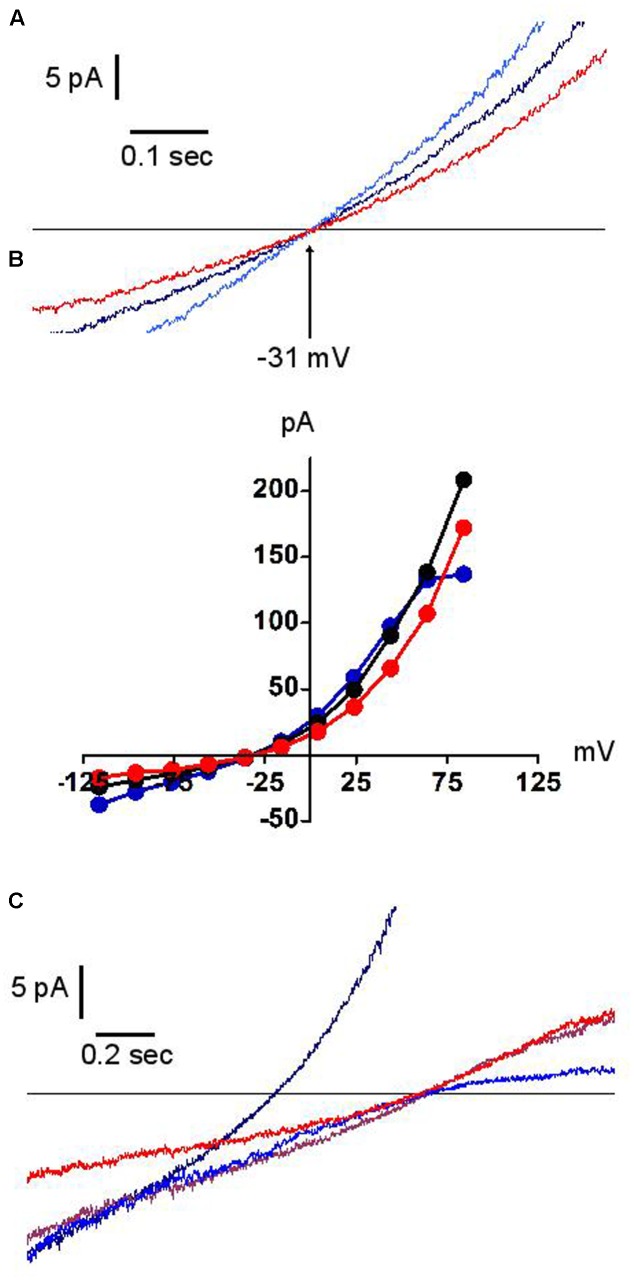
**Effects of external pH on the dialysis activated Cl^–^ current in HVCN1^–/–^ neutrophils (*n* = 3)**. Typical examples (*n* = 3). Black traces pH 7.3, blue traces pH 5.7, red traces pH 9.0. Experiments were carried out in solution set E (**Table 1**). **(A)** The reversal potential (–31 mV) is insensitive to pH. **(B)** Full IVs: pH has only minor effects on the current amplitude. **(C)** In low Cl^–^ concentration solution (10 mM), outward rectification is seen at high pH (red trace), inward rectification at low pH (blue trace). The brown trace represents pH 7.3, 10mM Cl^–^ solution. The black trace represents pH 7.3 at normal Cl^–^ concentration.

**Figure 6** illustrates the effect of external acidification (to pH 5.7) and alkalinisation (to pH 9.0) on the dialysis-activated Cl^-^ current. Focusing on the region of current reversal, we have been unable to distinguish any displacement of the reversal potential over four units of external pH change. **Figure 6** shows a representative example. The junction potential corrected reversal potential is at -30.5 mV (i.e., within 1 mV of its expected value on the basis of the Cl^-^ concentrations in external and pipette solutions). Its average displacement between external pH 5.7 and 9.0 was +0.75 mV (*n* = 3). Under the same conditions, the expected change in the reversal potential of an antiporter exchanging two Cl^-^ ion for one proton would be 65.7 mV, from -5.8 mV at pH 5.7 to -71.5 mV at pH 9.0. Our results clearly demonstrate that the channel under investigation is not of the proton antiporter type. While permeation is excluded, protons were not without effect: acidic external pH modestly potentiated the instantaneous and steady-state current except at high potential where the latter effect is counteracted by the fact that acidic pH also amplified the “sag” of the current (as shown in **Figure 5C**). Alkaline pH had a small inhibitory effect (**Figure 6B**).

In contrast, as expected, the reversal potential was largely shifted by the reduction of external Cl^-^. Whilst in such conditions its position remained insensitive to external pH, the current amplitude acquired a remarkably asymmetric pH dependence, with acidic pH reducing the outward current and alkaline pH reducing the inward current (**Figure 6C**). We have not explored the mechanistic implications of this observation.

### Selectivity

As is essential to the capacity of *I*_Cl_ swell in regulatory volume decrease and while being strongly outwardly rectifying, the dialysis activated Cl^-^ current in human and mouse neutrophils had a clear inward component, which allowed the direct observation of its reversal potential. After correction for junction potential, this reversal sat within 2 mV of the Nernst equilibrium potential for Cl^-^. A three point estimation of the equilibrium potential against the external Cl^-^ concentration was best fitted with a slope of 42 mV/decade.

Selectivity was verified in bi-ionic experiments, balancing an outward flux of Cl^-^ against the inward movements of fluoride, bromide, nitrate, gluconate and iodide ions. The mean (*n* = 3) relative permeabilities ranked as follows: I^-^ ≈NO_3_^-^ (1.3) > Br^-^ (1.21) > Cl^-^ (1) > F^-^ (0.71) >>> gluconate (0.16). This conformed to the Eisenmann’s anions sequence 1 that is generally observed with volume activated ion channels (Eisenman et al., 1983).

### The Dialysis-Activated Current Is Insensitive to Classical Neutrophil Activating Agents and Absent in Perforated Whole-Cell Recordings

Phorbol 12-Myristate 13-Acetate (PMA) or N-Formyl-L-methionyl-L-leucyl-L-phenylalanine (FMLP), (the latter with co-applied Cytochalasin B, 5 μg/ml) irreversibly activate neutrophils (McPhail and Snyderman, 1983; Segal et al., 1985) which involves major structural changes in the plasma membrane as the membranes of the cytoplasmic granules become incorporated into it when they degranulate to the exterior, which takes place at the same time as the burst of extracellular oxygen reduction via the NADPH oxidase. When applied in the “on cell” configuration prior to breakthrough, these agents were nevertheless unable to alter the properties of the dialysis activated current that breakthrough subsequently induces (**Figure 4**). While the insensitivity of the swell activated channel to the PMA activated protein kinase C has been documented in other contexts (Abdullaev et al., 2006), the lack of effect of both stimuli in neutrophils suggest that, if the channel has mechano-sensing properties, these are not modified by the relaxation that may accompany degranulation or the disruption of their cytoskeleton by cytochalasin B.

As these results were obtained in the presence of diphenyleneiodonium (DPI), they do not rule out a modulation of the dialysis-activated Cl^-^ current by the reactive oxygen products of the NADPH oxidase (Varela et al., 2004). However, exogenous H_2_O_2_ (1 mM) was without effect on the established dialysis activated current (data not shown).

As with human cells, this current was not apparent in the perforated whole cell configuration. Remarkably, this remained true when the pipette solution was of low ionic strength. Cytochalasin B, FMLP and the direct application of H_2_O_2_ remained unable to activate the current in that mode. It did, however, quickly emerge following mechanical breakthrough (data not shown).

### Identification of the Current Carrier As Being LRRC8A Dependent

**FIGURE 7:**
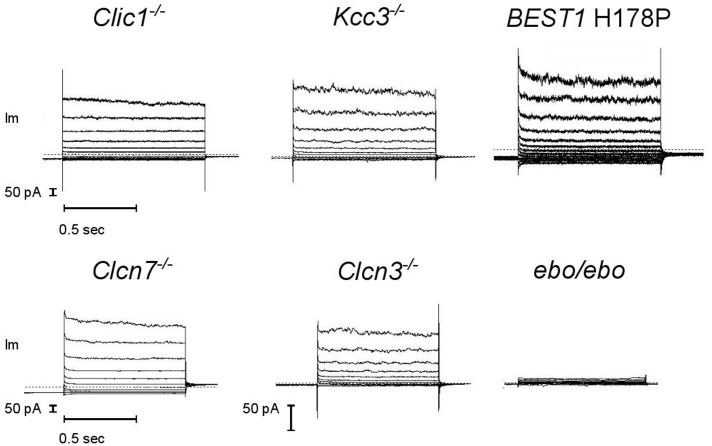
**A robust dialysis activated Cl^–^ current is recorded from mouse neutrophils lacking a range of Cl^–^ transport pathways but it is much reduced in LRRC8A deficient cells**. CLIC1^–/–^, KCC3^–/–^, CLCN3^–/–^ and *ebo/ebo* recordings are from bone marrow derived neutrophils, CLCN7^–/–^ recordings are from mouse blood neutrophils, BEST1 H178P recordings are from human neutrophils. Representative examples, *n* ≥ 3 for each subtype. Experiments were carried out using solution Set E (**Table 1**).

A number of Cl^-^ channels and transporters are expressed in neutrophils. We tested a few candidates for supporting the large current described above. Considering the possibility of degranulation, spontaneous or controlled, our genetic screening did include channels that are known to be primarily intracellular. It also considered electroneutral transporters to exclude the possibility of current arising from an alternative stoichiometry (Matsuda et al., 2010), or that of a functional coupling of these electrically silent transporters with a pore. We found the same dialysis activated Cl^-^ currents in bone marrow derived neutrophils from CLIC1, CLCN3, CLCN7 and KCC3 KO animals and in blood neutrophils from a Bestrophinopathy patient (Best1 H178P) (**Figure 7**, representative current traces). Our numbers of measurements were insufficient to be sure that in these cells current frequencies and amplitudes are indistinguishable from those of WT or control neutrophils.

**FIGURE 8:**
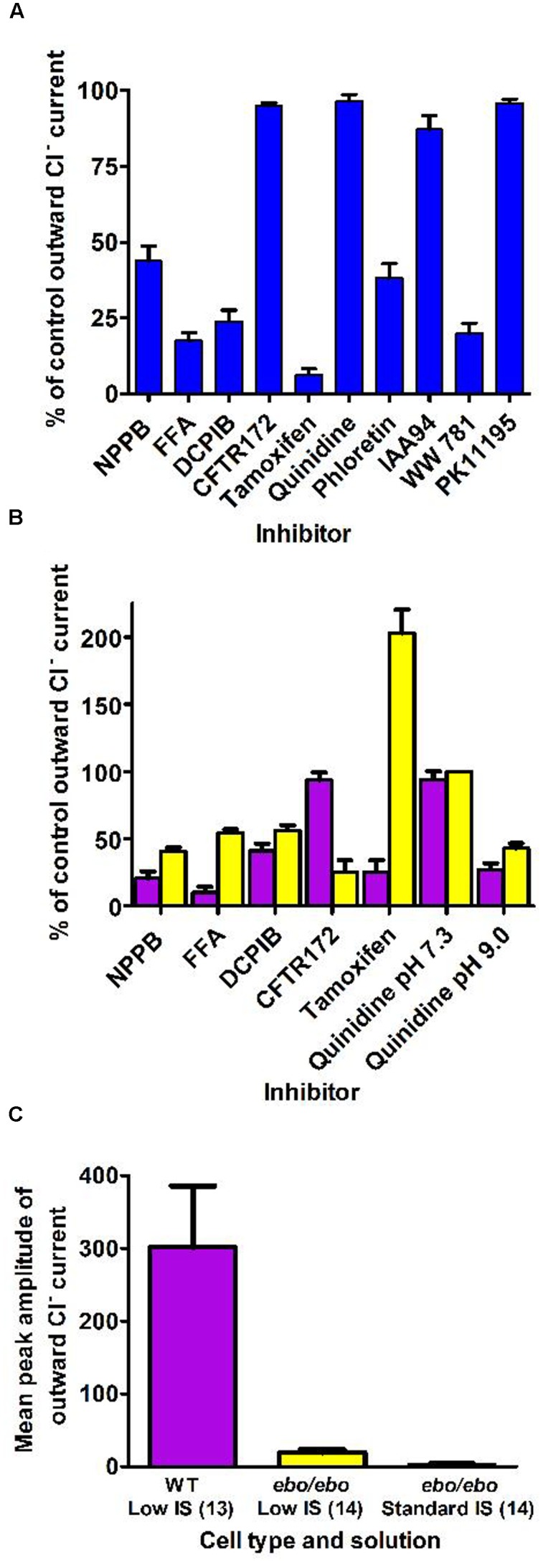
**(A)** Pharmacology of the dialysis activated current in HVCN1^–/–^ C57BL/6 mice neutrophils. Concentrations of inhibitors used (numbers of cells tested) were: NPPB 100 μM (14), FFA 200 μM (13), DCPIB 10 μM (4), CFTR-172 10 μM (4), tamoxifen 10 μM (4), quinidine 100 μM pH 7.3 (5), phloretin 200 μM (2), IAA94 50 μM (6), WW781 25 μM (5), PK11195 20 μM (4). The error bars represent standard error of the mean. **(B)** Pharmacology of the Cl^–^ current in WT BALB/c mice (purple bars) and of the residual Cl^–^ current in the *ebo/ebo* BALB/c mice (yellow bars). Concentrations of inhibitors used (number of WT cells, number of *ebo/ebo* cells used): NPPB 100 μM (9,9), FFA 200 μM (5,5), DCPIB 20 μM (8,9), CFTR-172 10 μM (4,2), quinidine 100 μM pH 7.3 (4,2), quinidine 100 μM pH 9.0 (4,2), tamoxifen 10 μM (4,6). The error bars represent standard error of the mean. **(C)** Ionic strength sensitivity: while a significant reduction of the outward Cl^–^ current recorded in low ionic strength intracellular solution is observed in *ebo/ebo* neutrophils compared to WT neutrophils, the residual Cl^–^ current in *ebo/ebo* neutrophils remains largely sensitive to ionic strength when in standard ionic strength solution.

By contrast, neutrophils of the *ebo/ebo* mice, which express a truncated version of the LRRC8A protein that is unable to form functional Cl^-^ channels (Platt et al., 2017), were found to exhibit drastically reduced Cl^-^ conductances. We opted to study this model of VSOR-deficiency rather than LRRC8A^-/-^ mice because the latter have a high perinatal mortality. The relative amplitudes of dialysis activated current in WT and in *ebo/ebo* dysfunctional neutrophils are compared in **Figure 8**, which also summarizes their pharmacology, as discussed below.

### Pharmacology

The pharmacology of the dialysis activated Cl^-^ current was investigated in HVCN1^-/-^ (C57BL/6) mouse neutrophils using CsCl or NMDG Cl^-^ based solutions. We tested a range of well-established Cl^-^ channel blockers on the current at its peak, or during the slow run-down phase that followed, at doses known to be maximally effective on other characterized Cl^-^ transporters. Their effects on the current level achieved at the end of 0.5 s pulses to +100 mV are summarized in **Figure 8A**. As observed on bovine pulmonary artery cells (Voets et al., 1996), the efficacy of quinidine was voltage dependent: only the unprotonated molecule appeared able to block the dialysis activated current of neutrophils.

A subset of blockers were also tested on the smaller current that was seen to persist in *ebo/ebo* mouse neutrophils. That the current seen in *ebo/ebo* mice neutrophils is carried by Cl^-^ was insured by the stringent selectivity of internal and external solutions (NMDG based, set E) and confirmed by its reduction in low external Cl^-^ with concomitant rightward shift of the current reversal potential [WT BALB/c cells: 32.9 ± 3 mV (*n* = 9); *ebo/ebo* cells: 28.39 ± 1.8 mV (*n* = 11)]. As shown on **Figure 8B, NPPB, FFA and DCPIB** were effective blockers of the current remaining in these LRRC8A deficient cells and the pH specificity of its block by quinidine replicates that seen in WT cells. Despite a much increased resolution for the detection of alternative Cl^-^ conductances in these cells, no sensitivity to the CFTR inhibitor was observed. Remarkably, the effects of tamoxifen on WT and *ebo/ebo* cells were divergent: As on HVCN1^-/-^ (C57BL/6) cells, tamoxifen causes a slow (mean delay to full inhibition ∼120 s), but profound, inhibition of the current in LRRC8A expressing BALB/c neutrophils. In contrast, a strong potentiation of the small persisting Cl^-^ current is observed in neutrophils from the *ebo/ebo* mice. In both cases, evidence of toxicity, manifest as sudden and large increases in non-specific conductance, was produced by tamoxifen after a delay. The effects quantified here were obtained before the occurrence of such artifacts and without a detectable change in the current reversal potential.

The outward current expressed by *ebo/ebo* neutrophils was clearly less rectifying than the major dialysis activated current. The ratio of conductances measured in 20 mV ranges centered on -80 and +85 mV were 11.1 ± 1.5 (*n* = 11) and 2.84 ± 0.23 (*n* = 15) for BALB/c WT and *ebo/ebo* neutrophils, respectively. A run up profile after breakthrough was still apparent. Surprisingly, we have observed that it also appeared to be amplified by a decreased ionic strength of the intracellular media: to allow a direct comparison with their effect on wild-type cells in which the dialysis activated current was promoted by internal solution of reduced ionic strength, the potency of blockers in *ebo/ebo* cells (**Figure 8B**) was investigated with the same internal solution. When we undertook to further qualify the remaining current with an internal solution of standard ionic strength in order to extend the significance of our results, we found the current in the *ebo/ebo* neutrophils current to be strongly reduced. Mean peak outward current with internal of standard ionic strength was only 16% percent of that seen in the same cells with a low-salt internal (**Figure 8C**) – there was no run up. Gluconate-sensitive activity or residual Cl^-^ current was either absent or limited to a few pA of current, occasionally consisting of resolved single channel activity. This suggests a relative sensitivity to ionic strength in *ebo/ebo* cells equal to that we observed in WT cells, and assumed typical of their dialysis activated current. While both the size and inherent variability of such activity complicated its analysis, it was clear that its reversal and amplitude were not affected enough by acidic (pH 6) or alkaline (pH 9) external media [respectively, inducing 3 mV (*n* = 9) positive and negative shift of its reversal] to imply an antiporter type of activity (which could also not account for the single channel activity that was occasionally resolved). Ionomycin, at up to 10 μM, also failed to induce or modulate the very small gluconate sensitive current that remains in *ebo/ebo* cells in standard ionic strength conditions.

### Functional Effects of LRRC8A Repression at the Vacuolar Level: pH and Volume of the Phagocytic Vacuole

Considering the highly debated role of Cl^-^ as an essential participant in neutrophil bactericidal activity, we measured the effect of tamoxifen and the consequences of LRRC8A deficiency on vacuolar pH and volume.

**FIGURE 9:**
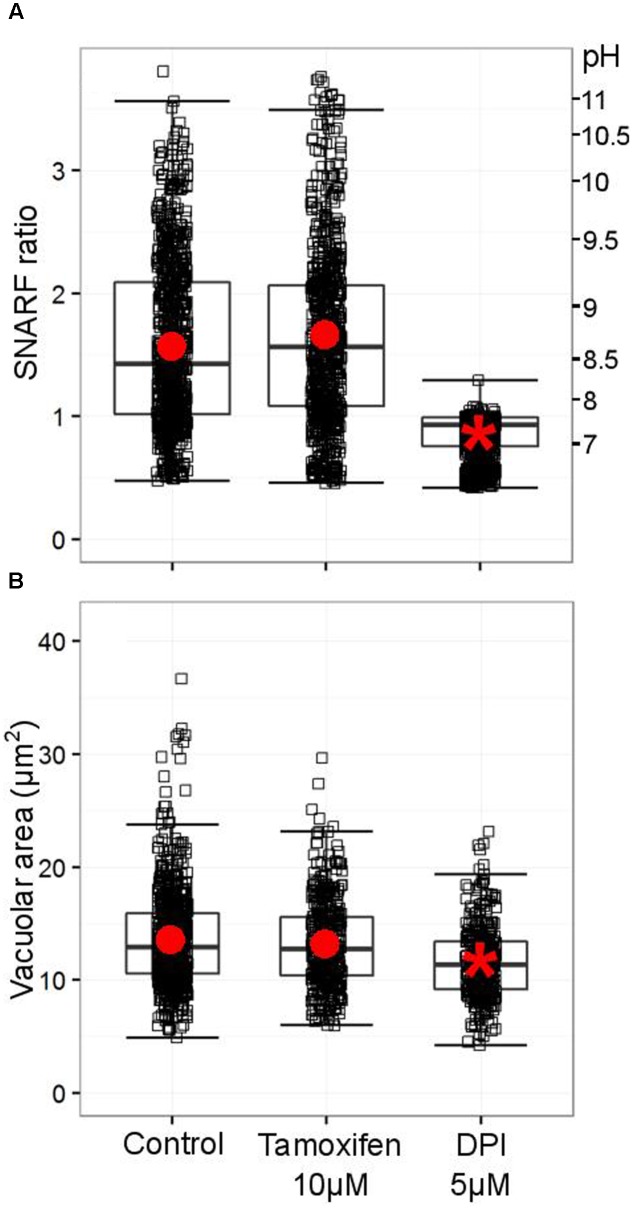
**Tamoxifen does not counteract the oxidase-driven alkalinization and swelling of the vacuole. (A)** Distribution SNARF fluorescence ratios measured in a minimum of 700 fully phagocytosed SNARF-labeled *Candida* by human neutrophils. Fluorescence ratios are indicated on the left-hand side, a corresponding pH scale is provided on the right-hand side (see Materials and Methods for calibration of SNARF fluorescence ratio to pH). **(B)** Distribution of areas measured in a minimum of 340 individual vacuoles incorporating a single *Candida*. Boxes show medians and interquartile ranges of data, compiled from three independent experiments. Red circles locate the mean values, the asterisk locating the mean for DPI denotes statistical significance from the control *p* < 0.05.

As originally described by Segal et al. (1981) and recently confirmed by Levine et al. (2015), the pH of the vacuole rises rapidly after phagocytosis of the microbe. Foote et al. (2017) have demonstrated that a range of Cl^-^ channel blocker can reduce this alkalinisation, but tamoxifen, which strongly blocks the swell activated channel, was clearly unable to do so (**Figure 9A**). It was also without effect on the volume of the phagocytic vacuole (**Figure 9B**).

**FIGURE 10:**
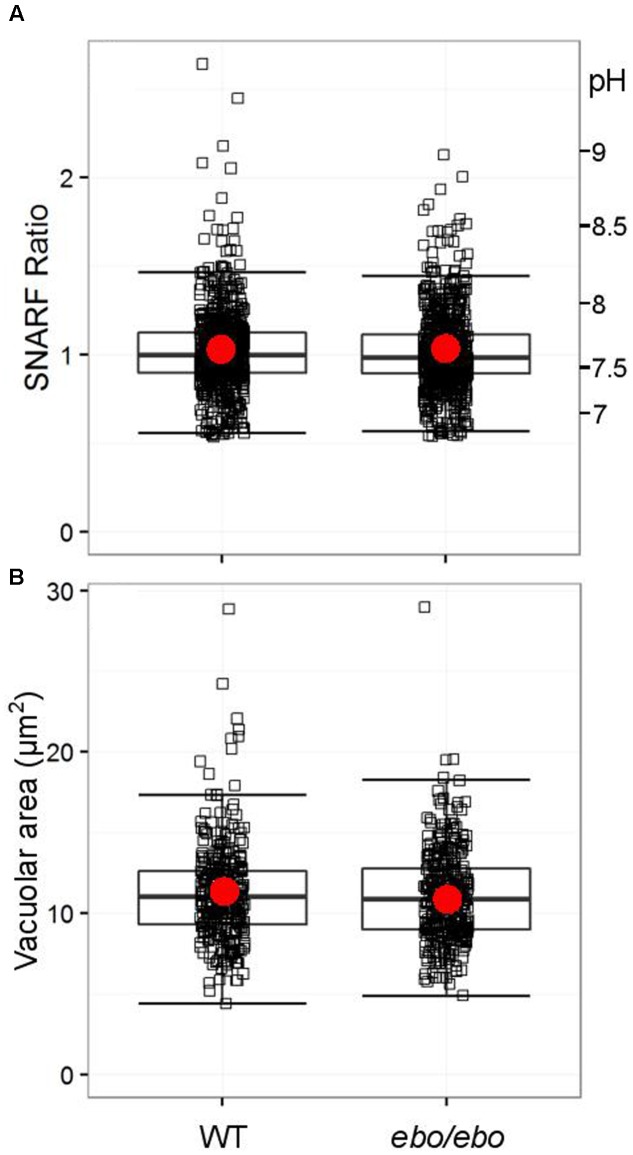
**(A)** Distribution of SNARF fluorescence ratios measured on fully phagocytosed SNARF labeled *Candida* by WT and *ebo/ebo* neutrophils (733 and 735 *Candida* measured, respectively, compiled from three independent experiments). **(B)** Distribution of areas of vacuoles incorporating a single *Candida* in WT and *ebo/ebo* neutrophils (320 and 354 vacuoles measured, respectively, compiled from three independent experiments). Boxes show medians and interquartile ranges, compiled from three independent experiments. Red circles locate the mean values.

The ineffectiveness of tamoxifen suggests that the dialysis activated current, shown above to rely on the expression of LRRC8A in neutrophils, is not involved in pH-setting ion exchange at the phagosomal membrane. We verified that *ebo/ebo* neutrophils have a normal vacuolar pH (**Figure 10A**) and volume (**Figure 10B**), increases of which are dependent upon oxidase activity.

**FIGURE 11:**
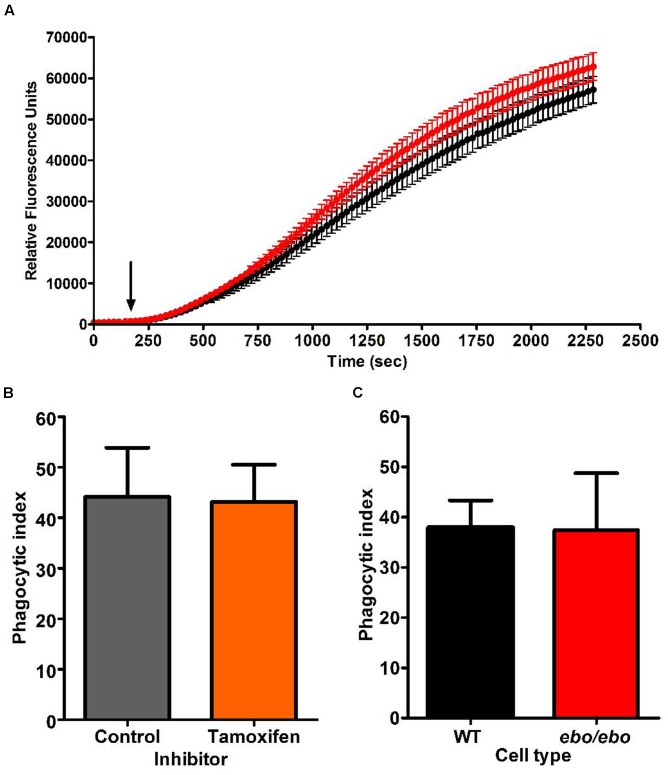
**(A)** H_2_O_2_ production by PMA stimulated WT (black trace) and *ebo/ebo* (red trace) neutrophils. The mean ± SEM is displayed from two independent experiments involving triplicate measures. The arrow marks the timing of PMA injection. **(B)** Tamoxifen (10 μM) does not affect the phagocytosis of opsonised *Candida* (*n* = 2, ± SEM) as measured by phagocytic index (**Table 1**) in human neutrophils. **(C)** There is no difference in the phagocytosis by *ebo/ebo* neutrophils compared to WT neutrophils (*n* = 3, ± SEM).

As we experienced difficulties in gathering enough *ebo/ebo* neutrophils to reliably assess their oxygen consumption upon phagocytosis of *Candida*, we were constrained to evaluate their oxidase activity at the plasma membrane by measuring hydrogen peroxide produced in response to the soluble stimulus PMA. Under these circumstances, *ebo/ebo* neutrophils were as competent as WT cells (*n* = 2) (**Figure 11A**).

In the microglia associated BV2 cells, Cl^-^ channel blockers (FFA and NPPB) were shown to antagonize phagocytosis (Furtner et al., 2007). In human neutrophils, however, we confirmed the ineffectiveness of tamoxifen in that respect (as implied by Moreland et al., 2006) (**Figure 11B**). Under our experimental conditions [average cell density 0.52/(10μm)^4^ and 1.22 *Candida*/cell], the phagocytic capacity of *ebo/ebo* neutrophils was not found to differ from that of WT cells (**Figure 11C**).

## Discussion

This investigation was directed toward identifying a Cl^-^ channel that participates in compensating the charge induced across the wall of the phagocytic vacuole by the passage of electrons through the NADPH oxidase, NOX2.

Activity of the neutrophil NOX2 induces an alkalinisation of the vacuole that is important for activating the antimicrobial activity of the neutral proteases. This alkalinisation occurs because part of the oxidase induced charge is compensated by ions other than protons, cations passing into the vacuole and/or anions passing out (Levine et al., 2015).

We have recently demonstrated that Cl^-^ channel blockers prevent this alkalinisation, implicating them in charge compensation (Foote et al., 2017). Others have previously linked specific Cl^-^ channels to the oxidase, CFTR (Painter et al., 2006; Aiken et al., 2012) and KCC3 (Sun et al., 2012) bringing Cl^-^ into the vacuole, and CLCN3 (Moreland et al., 2006) exporting it. We have investigated mouse neutrophils lacking these channels, and found no alteration of vacuolar pH or volume (Foote et al., 2017). We have therefore used patch clamp studies to determine whether we could find relevant currents.

The membrane surrounding the phagocytic vacuole is formed from an invagination of the plasma membrane, and the NADPH oxidase drives electrons into the vacuole, which would be compensated by Cl^-^ passing from the vacuole into the cytoplasm, and then possibly expelled from the cell. This is compatible with the observed very significant efflux of Cl^-^ from phagocytosing neutrophils (Busetto et al., 2007).

The predominant Cl^-^ current that we found was outwardly rectifying and conformed to the physical and pharmacological characteristics of a “swell-activated” current. However, it would be functionally disadvantageous for the channel to be opened by the considerable mechanical deformation of the plasma membrane during its varied physical functions that include migration, diapedesis, phagocytosis and finally the oxidase-dependent swelling of the phagosomal compartment. In agreement with an earlier reassessment by Voets et al. (1999) we demonstrate that membrane stress or tension do not appear to constitute the primary activator of that Cl^-^ pathway; it rather senses and responds to the ionic strength of the intracellular medium at constant osmolarity and Cl^-^ driving force. Such isometric activation of a “swell like” Cl^-^ efflux pathway has been previously documented in a number of other cells (Wang et al., 1996; Nilius et al., 1998; Maeno et al., 2000).

Initially we studied neutrophils from mice lacking CLCN3 and KCC3, and the effect of the CFTR inhibitor on the presence of this Cl^-^ current and found it to be normal. Subsequently, the role of LRRC8A in the classical swell-activated channel was identified (Qiu et al., 2014; Voss et al., 2014), so we examined these currents in neutrophils from *ebo/ebo* mice. This is a spontaneous mouse mutant ébouriffé (tousled) that harbors a 2-bp frameshift mutation in *LRRC8A* that truncates the 15 terminal LRRs. The mutation does not affect protein expression but drastically diminishes VRAC activity. We chose to use this mutant because *LRRC8* KO animals are very difficult to breed (Kumar et al., 2014).The current we found to be activated in neutrophils by internal solutions of reduced ionic strength was markedly diminished in neutrophils from these mice, identifying the current we had observed as that through the swell-activated channel containing LRRC8A.

Interestingly, we found a small Cl^-^ current in these *ebo/ebo* mice that was sensitive to ionic strength. This could be due to a small residual current through the mutant LRRC8A. In favor of this possibility is the fact that the *ebo/ebo* mice had a slightly different phenotype from the LRRC8A KO mice. *Ebo/ebo* mice share features with LRRC8A^-/-^ mice that include curly hair, infertility, reduced longevity, and kidney abnormalities. However, in contrast to LRRC8A^-/-^ mice, *ebo/ebo* mice have normal T-cell development and function and intact antibody response to T-dependent antigen (Platt et al., 2017). It could be that a small residual current through the mutant LRRC8A is sufficient for the function of immune cells but insufficient to prevent the morphological abnormalities seen in both mice. On the other hand, a multitude of Cl^-^ channels have been proposed as responding to cell swelling in protocols that are likely to generate a dilution of the cytoplasmic salts, and the residual Cl^-^ current we found in the *ebo/ebo* mice displayed an altered rectification, and an alternative pharmacology, characterized by a potentiating effect of tamoxifen. Prior comparison of VSOR activity in LRRC8A^-/-^ and *ebo/ebo* T cells found no significant difference in the residual current density measured at -80 mV (Platt et al., 2017). However, when analyzed at +80 mV, the residual VSOR activity was slightly higher in *ebo/ebo* T cells than in LRRC8A^-/-^ T cells. This suggests that residual VSOR activity can be induced under what are likely to be non-physiological conditions in T cells. It is possible that the abnormal tertiary structure of the mutant LRRC8A is responsible for the altered rectification and altered pharmacology we observed in the small residual current.

We demonstrate that these *ebo/ebo* neutrophils are competent phagocytes and that they form vacuoles whose size and pH are not distinguishable from that of their wild type counterparts, indicating that the LRRC8A based Cl^-^ conductance of neutrophils is not essential for phagocytosis and homeostasis of the vacuole.

Our data discount a role for the major, if not only, swell activated conductance, in vacuolar homeostasis in neutrophils. Given the extremely small plasma membrane based Cl^-^ conductance that is independent of that pathway, the participation of Cl^-^ channels in charge compensation at the vacuolar level may rely on a specific vacuolar subtype of channel.

## Author Contributions

AS, PB, AL, and JF contributed to the conception and design of the study. CP, JC, FB, and RG made the *ebo/ebo* mice. PB conducted the patch clamp electrophysiology, JF and AL the cellular assays. All took part in manuscript preparation.

## Conflict of Interest Statement

The authors declare that the research was conducted in the absence of any commercial or financial relationships that could be construed as a potential conflict of interest. The reviewer PI and handling Editor declared their shared affiliation, and the handling Editor states that the process nevertheless met the standards of a fair and objective review.

## Footnotes

1 http://maxchelator.stanford.edu

2 http://www.pco.de

3 http://imagej.nih.gov/

4 http://www.pco.de
